# Iron-Reduced Graphene Oxide Core–Shell Micromotors Designed for Magnetic Guidance and Photothermal Therapy under Second Near-Infrared Light

**DOI:** 10.3390/pharmaceutics16070856

**Published:** 2024-06-25

**Authors:** Orlando Donoso-González, Ana L. Riveros, José F. Marco, Diego Venegas-Yazigi, Verónica Paredes-García, Camila F. Olguín, Cristina Mayorga-Lobos, Lorena Lobos-González, Felipe Franco-Campos, Joseph Wang, Marcelo J. Kogan, Soledad Bollo, Claudia Yañez, Daniela F. Báez

**Affiliations:** 1 Departamento Química Farmacológica y Toxicológica, Facultad de Ciencias Químicas y Farmacéuticas, Universidad de Chile, Sergio Livingstone #1007, Independencia, Santiago 8380492, Chile; orlando.donoso@ug.uchile.cl (O.D.-G.); ana.riveros@ciq.uchile.cl (A.L.R.); mkogan@ciq.uchile.cl (M.J.K.); sbollo@ciq.uchile.cl (S.B.); 2 Advanced Center for Chronic Diseases (ACCDiS), Universidad de Chile, Sergio Livingstone #1007, Independencia, Santiago 8380492, Chile; cristina.mayorga@ug.uchile.cl; 3 Instituto de Química Física Blas Cabrera, Consejo Superior de Investigaciones Científicas (CSIC), Serrano 119, 28006 Madrid, Spain; jfmarco@iqfr.csic.es; 4 Departamento de Química de los Materiales, Facultad de Química y Biología, Universidad de Santiago de Chile, Libertador Bernardo O’Higgins #3363, Estación Central, Santiago 9170022, Chile; diego.venegas@usach.cl; 5 Centro para el Desarrollo de La Nanociencia y la Nanotecnología (CEDENNA), Universidad de Santiago de Chile, Libertador Bernardo O’Higgins #3363, Estación Central, Santiago 9170022, Chile; vparedes@unab.cl; 6 Departamento de Ciencias Químicas, Facultad de Ciencias Exactas, Universidad Andrés Bello, República 275, Santiago, Santiago 8370146, Chile; 7 Escuela de Medicina, Universidad de Talca, Talca 3460000, Chile; camila.olguinc@usach.cl; 8 Cellular Communication Laboratory, Center for Studies on Exercise, Metabolism and Cancer (CEMC), Institute of Biomedical Sciences (ICBM), Faculty of Medicine, University of Chile, Santiago 8380492, Chile; llobos@udd.cl; 9 Center for Regenerative Medicine, Institute for Sciences and Innovation in Medicine, Facultad de Medicina, Clínica Alemana Universidad del Desarrollo, Santiago 7610658, Chile; 10 Research Group in Alternative Methods for Determining Toxics Effects and Risk Assessment of Contaminants and Mixtures (RiskTox), Laboratory of Food Chemistry and Toxicology, Faculty of Pharmacy, University of Valencia, 46100 Valencia, Spain; felipe.franco@uv.es; 11 Department of Nanoengineering, University of California San Diego, La Jolla, CA 92093, USA; josephwang@ucsd.edu; 12 Centro de Investigación de Procesos Redox, CIPRex, Facultad de Ciencias Químicas y Farmacéuticas, Universidad de Chile, Sergio Livingstone #1007, Independencia, Santiago 8380492, Chile; 13 Departamento de Química Orgánica y Fisicoquímica, Facultad de Ciencias Químicas y Farmacéuticas, Universidad de Chile, Sergio Livingstone #1007, Independencia, Santiago 8380492, Chile

**Keywords:** core–shell micromotors, magnetic guidance, photothermal effect, reduced graphene oxide, photothermal therapy, cancer

## Abstract

Core–shell micro/nanomotors have garnered significant interest in biomedicine owing to their versatile task-performing capabilities. However, their effectiveness for photothermal therapy (PTT) still faces challenges because of their poor tumor accumulation, lower light-to-heat conversion, and due to the limited penetration of near-infrared (NIR) light. In this study, we present a novel core–shell micromotor that combines magnetic and photothermal properties. It is synthesized via the template-assisted electrodeposition of iron (Fe) and reduced graphene oxide (rGO) on a microtubular pore-shaped membrane. The resulting Fe-rGO micromotor consists of a core of oval-shaped zero-valent iron nanoparticles with large magnetization. At the same time, the outer layer has a uniform reduced graphene oxide (rGO) topography. Combined, these Fe-rGO core–shell micromotors respond to magnetic forces and near-infrared (NIR) light (1064 nm), achieving a remarkable photothermal conversion efficiency of 78% at a concentration of 434 µg mL^−1^. They can also carry doxorubicin (DOX) and rapidly release it upon NIR irradiation. Additionally, preliminary results regarding the biocompatibility of these micromotors through in vitro tests on a 3D breast cancer model demonstrate low cytotoxicity and strong accumulation. These promising results suggest that such Fe-rGO core–shell micromotors could hold great potential for combined photothermal therapy.

## 1. Introduction

Micro/nanomotors (MNM) are devices with the ability to generate autonomous movement through the conversion of several forms of stimulation, such as chemical reagents, light, ultrasound frequencies, and magnetic fields, among others [1,2,3,4]. To date, a large number of MNM with diverse morphologies and compositions have been geared toward biomedical and environmental applications [5,6,7,8]. In biomedicine, they have demonstrated outstanding performance in detecting or treating primary diseases such as cancer [9,10,11,12,13]. This ability is due to their continuous movement, which enhances their interaction with target molecules [14,15], facilitates drug delivery [16], improves the accumulation rate in complex biological environments [17], and enables penetration into tissues [18,19,20,21].

When designing and developing this kind of structure, it is necessary to select the driving stimuli, a crucial aspect when considering their introduction into the living body [6,22,23,24]. In this sense, magnetic motion has shown advantages compared to other kinds of stimuli, including external motion control, the possibility of tracking, the avoidance of fuel usage, as well as features like reconfigurability and recyclability [1,3,8,16,25,26,27,28,29,30,31,32,33,34]. Several efforts have been made to advance the operation of magnetic MNM in body fluids and whole organisms. Examples include hybrid micromotors using sperm flagellum to swim against flowing blood [35] or a magnetic stem cell spheroid that can interface with the human body navigating the bile duct [1]. However, this kind of biological component could make their clinical translation difficult. Among the simplest magnetic materials used for magnetic micromotors, nickel, iron, and their derivatives are the most reported [16,26,28]. However, iron and iron oxide-based materials are preferred because they present better biocompatibility instead of the potential negative effects that nickel can generate in animal and human bodies [36,37,38,39,40,41].

Photothermal therapy (PTT) is a noninvasive anticancer strategy that treats locally different types of tumors, promoted by materials known as photothermal agents (PTAs). These PTAs present the property of absorbing light and converting it into heat through different mechanisms [42]. The locally high temperature generates hyperthermia, leading to cancerous cell destruction, with minimum damage to normal tissue cells. Some nanomaterials, such as copper and gold nanoparticles [43,44] and some organic polymer nanoparticles [42,45,46], have been described as efficient heaters for photothermal therapy [25]. Notably, graphene-based nanomaterials like graphene oxide (GO) and reduced graphene oxide (rGO) [47,48] also share this essential property but also possess a large specific surface area, efficient adsorption properties, and a high absorption of light from the first and second NIR regions [49,50,51]. This aspect is essential since PTT triggered by light from the second NIR window (1000–1700 nm) has attracted significant attention [52] due to its ability to penetrate deeper into tissues. Also, there is the possibility of using a laser source with higher power intensities, causing negligible damage to the treated tissue. These properties make the therapy more effective when tumors are located deep within tissues.

Graphene-based micromotors have been previously developed as active systems to capture [31], transfer, and remove different kinds of analytes [53,54] and detect cancer biomarkers [32]. In these kinds of micromotors, the graphene component has been mostly used as the platform to support the recognition probe or to enhance the movement of light-driven micromotors [55,56]. However, their applicability as photothermal agents to treat cancer using hyperthermia has been little explored. Herein, the main purpose of this work was to develop a kind of micromotor to use as a magnetically guided photothermal agent. To that end, we carried out the micromotor preparation through the electrochemical template-assisted deposition method, placing rGO first to support the iron component and the loading of an anticancer drug. This arrangement resulted in a core–shell microtube with a core of iron nanoparticles and an outer layer of reduced graphene oxide (Fe-rGO). The exhaustive morphological characterization of Fe-rGO micromotors through scanning electron microscopy (SEM) confirmed the core–shell microtubular shape. The X-ray powder diffraction (XRD), X-ray photoelectron spectroscopy (XPS), and ^57^Fe Mössbauer measurements demonstrated that the prepared Fe-rGO core–shell micromotors presented a soft ferromagnetic behavior attributed to the zero-valent iron nanoparticles in the core that allowed the controlled magnetic movement. The photothermal capacity of Fe-rGO micromotors, evaluated through irradiation with an 808 and 1064 nm NIR laser, confirmed in the first place that increased temperatures are obtained when using laser light from the second near-infrared window. Then, a high photothermal conversion efficiency, as well as the fast release of doxorubicin (DOX), was achieved under the application of NIR irradiation at 1064 nm. Additionally, preliminary viability assays on the MDA-MB-231 breast cancer cell line demonstrated that Fe-rGO micromotors presented no cytotoxicity for the three concentrations assessed. Moreover, strong accumulation was observed when Fe-rGO micromotors were magnetically stirred toward a 3D breast cancer tumoroids model.

## 2. Materials and Methods

### 2.1. Materials and Reagents

Our lab previously synthesized and characterized GO through a modified Hummer’s method, and this reagent was dry-stored [50]. The alumina slurry was purchased from G. Busch & Cia. Ltd. (Valparaiso, Chile). Sulfuric acid (H_2_SO_4_) > 98%, MW: 98.08 g·mol^−1;^ sodium sulfate (NaSO_4_) > 99.0%, MW: 142.04 g·mol^−1^; iron chloride (FeCl_2_·4H_2_O) > 98%, MW: 198.81 g·mol^−1^; ammonium chloride (NH_4_Cl) > 99.995%, MW: 53.49 g mol^−1^; iron sulfate (FeSO_4_·7H_2_O) > 99.5%, MW: 278.01 g·mol^−1^; and potassium chloride (KCl) > 99.5%, MW: 74.56 g·mol^−1^ were purchased from Merck (Darmstadt, Germany). Cyclopore polycarbonate membranes (47 mm) with 5 µm tubular-shaped pore diameters (catalog No 7060-4710) were purchased from Whatman (Little Chalfont, UK). Methylene chloride (CH_2_Cl_2_) > 99.8%, MW: 84.93 g·mol^−1^; isopropanol ((CH_3_)_2_CHOH) > 99.5%, MW: 60.10 g·mol^−1^; ethanol (CH_3_CH_2_OH) > 99.2%, MW: 46.07 g·mol^−1^; doxorubicin hydrochloride (C_27_H_29_NO_11_·HCl) > 98%, MW: 579.98 g·mol^−1^; and phosphate-buffered solution (PBS) tablets < 99% were obtained from Sigma Aldrich (Saint Louis, MO, USA). Ultrapure water was used to prepare all solutions.

### 2.2. Solutions

GO was prepared with 2.0 mg mL^−1^ in 20 mL of sulfuric acid (0.1 M) by ultrasonication for 15 min. Then, 0.014 g of Na_2_SO_4_ was added to the solution and manually stirred. The iron solution was prepared with 0.84 g of FeCl_2_·4H_2_O in 20 mL of NH_4_Cl (0.37 M), and then 5.0 g of FeSO_4_·7H_2_O was added to the solution and manually shaken. This solution is freshly prepared when needed.

### 2.3. Preparation of Micromotors

The electrochemical synthesis of microtubes was conducted by an EmStat4s *HR* potentiostat (PalmSens, Houten, The Netherlands); a Pt wire and an Ag/AgCl (3 M KCl) electrode were used as auxiliary and reference electrodes, respectively. A polycarbonate membrane of double truncated cone pore in shape (5.0 µm diameter) was sputtered on one side with a gold film of 35 nm and used as a template and working electrode. The sputter was performed using Luxor^Au^ Sputter Coater equipment under air at room temperature.

First, the membrane was assembled into a customized Teflon cell where 30 mL of GO (0.1 mg mL^−1^) solution was added to the cell and electrochemically reduced over the wall pores of the membrane at room temperature using cyclic voltammetry (CV, over +0.30 to –1.50 V, at 50 mV s^−1^, for ten cycles). Subsequently, the iron component was electrodeposited over the surface of rGO by applying, amperometrically, a potential of −0.95 V at different times (900, 2000, and 2500 s) to reach a sufficient charge that allows the microtubular shape. The microtubes were released from the template membrane by polishing the gold layer through a mechanical polish with a 5.0 µm alumina slurry. Then, the membrane was dissolved three times in methylene chloride for 10 min by manual stirring. Then, the microtubes were collected by decantation. Afterward, successive washes with isopropanol followed by ethanol and ultrapure water (three times) were performed, with a 3 min centrifugation at 3835× *g* between each wash. All microtubes were stored in ultrapure water at 4 °C when not in use. The template preparation method resulted in reproducible micromotors.

### 2.4. Photothermal Conversion Efficiency of Micromotors

The photothermal conversion efficiency of 109, 217, and 434 μg mL^−1^ synthesized Fe-rGO micromotor solutions was determined in a closed dark chamber by using 808 nm (350 mW) and 1064 nm (200, 300, 350, 400 and 500 mW) lasers (Power Technology, New York, NY, USA), located 20 cm from the sample. The sample was placed in a 9 mm diameter and 28 mm length tube. The temperature increase was monitored using an infrared thermographic camera E8-XT—Handheld Infrared Camera (Teledyne FLIR, Wilsonville, OR, USA) with an accuracy of ±0.1 °C, located 20 cm from the sample. The temperature and thermography images were recorded every 60 s, during a total time interval of 20 m. Ultrapure water was irradiated as a solvent control.

### 2.5. Loading Doxorubicin on Micromotors

To load doxorubicin (DOX) onto Fe-rGO micromotors, 350 μL of 217 μg mL^−1^ Fe-rGO micromotors dispersed in ultrapure water were magnetized, followed by the removal of the supernatant. Subsequently, the supernatant was substituted with 350 μL of 10 μM of DOX in PBS at pH 7.4, which were then added to the Fe-rGO and stirred at 200 rpm for 24 h at room temperature, protected from light. Hereafter, the dispersion of Fe-rGO/DOX was magnetized, and the supernatant was collected and replaced with PBS 7.4. The DOX supernatant was quantitatively analyzed through a calibration curve using the Beer–Lambert law method. Accordingly, a series of standard stock DOX solutions ranging from 0.5 to 25 μM were prepared. Subsequently, the concentration of DOX was assessed by measuring absorbance at 490 nm, both before and after the interaction with the micromotors, using a Synergy Mx Microplate Reader SMA (Byotek, Winooski, VT, USA) equipped with a high-energy xenon flash lamp. The loading capacity of micromotors was then calculated using the following Equation (1):

(1) $$Loading\;capacity\;(\%):\;\frac{\;amount\;of\;DOX\;loaded\;(µg)}{amount\;of\;micromotors\;(µg)}\times 100$$

### 2.6. Micromotor Photothermal-Driven Release of Doxorubicin

The release of DOX was carried out at ambient temperatures. A 1064 nm laser with 500 mW of power intensity irradiated the Fe-rGO/DOX dispersion for 3, 5, 10, and 15 min. After irradiation, the dispersion was magnetized, and the supernatant was mixed with the same volume of a known concentration DOX solution for quantitative analysis before and after NIR laser irradiation. The concentration of DOX in the supernatant was determined by spectrophotometry, using the Beer–Lambert law method and the calibration curve previously constructed.

### 2.7. Cell Lines and Cell Culture

In general, for the cell culture and all biological evaluation, Fe-rGO micromotors were sterilized using 20 min exposure to ultraviolet light in a biosafety cabinet. Then, the micromotors were always loaded and handled under sterile conditions and in a cell culture cabinet. Human breast cancer cell line MDA-MB-231 was obtained from American Type Culture Collection (ATCC, Manassas, VA, USA, HTB-26TM) and cultured in Dulbecco’s Modified Eagle Medium (DMEM)/F12 medium (Gibco, Carlsbad, CA, USA) supplemented with 10% fetal bovine serum (FBS; Biological Industries, Kibbutz Beit-Haemek, Israel), 100 IU/mL penicillin, 0.1 mg/mL streptomycin and 0.05 mg/mL gentamicin (Gibco). The cells were cultured at 37 °C and 5% CO_2_.

### 2.8. Cell Viability MTT Assay

Cell viability was assessed by using the 3-[4,5-dimethylthiazol-2-yl]-2,5diphenyl-tetrazolium bromide (MTT) (Promega, Fitchburg, WI, USA) assay. Briefly, MDA-MB-231 cells were seeded in pretreated 96-well plates at 3000 cells/well and allowed to attach at 37 °C and 5% CO2 for 24 h. Then, the medium was removed, and a fresh medium containing the treatment solutions was added and incubated. At 24, 48, and 72 h post-treatment, 20 µL/well of 5 mg ml^−1^ MTT was added and incubated for 2 h at 37 °C. Dimethyl sulfoxide (DMSO) was then added (150 µL/well) and incubated for 10 min at room temperature. Absorbance was measured at 570 nm in an Epoch microplate reader (Biotek, Winossky, VT, USA), and the cell viability was calculated compared to a nontreatment control as a live control. Statistical analysis was performed using GraphPad Prism V8.0.1. All the experimental data obtained using the MTT assay are expressed as means ± standard deviations and were analyzed using a non-parametric one-way ANOVA to calculate the significance level of the experimental data (*n* = 3). The differences were considered statistically significant at *p* ≤ 0.05.

### 2.9. Tumoroid or 3D Model Formation Assay

MDA-MB-231 breast cancer cells were seeded on sterile 2% agar-covered plates (6-well plates), supplemented with mammary epithelial cell growth culture medium (at least 1.5 mL of MEGM™ (Lonza, Basel, Switzerland, cat. CC-3151) supplemented with EGF 25 ng/mL, hydrocortisone 0.5 g/mL, insulin 5 µg/mL (Lonza, cat. CC-4136) and bFGF 25 ng /mL (Invitrogen, Waltham, MA, USA, cat. PHG0026). Tumoroids (tumor spheroids or a 3D cancer model) were grown at 37 °C and 5% CO_2_ for 14 days. The cell culture medium was not renewed during the 14 days of the experiment, and the formation of spheres was visually recorded by photography using the Micrometrics SE Premium 4.5.1 software in a Nikon Eclipse TS100 inverted microscope. After 14 days, the cell culture medium with the spheres was extracted from the well and passed through a 70 µm filter (BD Falcon, Franklin Lakes, NJ, USA, cat. 352350). The spheres retained on the filter were recovered and plated on a 12-well adhesion plate (Corning, Corning, NY, USA, cat. 3512).

### 2.10. Magnetic Motion

To magnetically operate the Fe-rGO-micromotors, a drop of 3 µL of the Fe-rGO micromotor solution, dispersed in ultrapure water or cellular medium (with or without tumoroids), was placed on a glass slide. Then, a magnetic field of 267 mT was applied by placing a small magnet away from the glass slide without changing the distances. By changing the direction of the magnet, the micromotors followed the orientation of the magnetic field. The motion of micromotors was registered using an inverted optical microscope (Olympus CKX41 equipped with a Nikon camera DS-Fi 2), and the Tracker AnalySIS 1.0 ^®^ software was used for the tracking analysis.

### 2.11. Instruments and Characterization

Scanning electron microscopy (SEM) images and energy dispersive X-ray (EDX) analyses were obtained using a Field Emission-SEM Inspect F50, FEI.

X-ray powder diffraction (XRD) was performed with a Phillips diffractometer operated at 30 kV and 20 mA, working in the Bragg–Brentano configuration and using Cu Kα radiation.

XPS data were recorded using a PHOIBOS-150 hemispherical analyzer (Specs), Al Kα radiation, and a constant pass energy of 20 eV under a base pressure lower than 5 × 10^−9^ mbar. The binding energy scale was referenced to the main contribution to the C 1s spectrum, which was set at 284.6 eV.

^57^Fe Mössbauer spectra were recorded at room temperature in the transmission mode using a conventional constant acceleration spectrometer and a ^57^Co(Rh) source. The velocity scale was calibrated using a 6 µm thick iron metallic foil, and the chemical isomer shifts were referred to the centroid of the spectrum of α-Fe at room temperature.

The magnetic behavior of the samples was measured using a vibrating sample magnetometer (VSM) (Quantum Design, model PPMS Dynacool). The magnetic properties of the magnetic micromotors were obtained from dry samples by VSM. The magnetization measurements were obtained by applying a variable magnetic field of ±90 kOe at 298 K.

## 3. Results

### 3.1. Characterization of Fe-rGO Core-Shell Micromotors

The Fe-rGO core–shell micromotors were prepared through the electrochemical template-assisted method, using an electrically conductive membrane with tubular-shaped pores as a template. Following our previously reported method, the electrochemical deposition of graphene oxide was the first step in forming the microtube [32]. Supplementary Figure S1a shows the consecutive cyclic voltammograms for the reduction of GO. The first scan shows a large reduction peak at −0.88 V. The main reduction of GO, which occurs in this scan, was attributed to the reduction of oxygen moieties, such as ketones, hydroxyls, aldehydes, and carboxylic acids [50]. Then, the rGO layer supported the electrodeposition of the iron component. Supplementary Figure S1b illustrates the i-t curves obtained during the electrodeposition of iron under varying conditions: a potential pulse of −0.95 V vs. Ag/AgCl applied for 900 (blue curve), 2000 (red curve), or 2500 s (black curve). Despite the differences in deposition duration, consistent electrochemical behavior is observed initially across all three experimental settings. Initially, a decrease in current intensity was attributed to the nucleation process of iron nanoparticles [57], followed by subsequent growth until reaching a plateau, indicating a steady state over time. The associated charges for these conditions were approximately 46.5, 82.4, and 167 C, respectively. After finishing the synthesis process, the microtubes were released from the template by manually polishing and dissolving the membrane with a gradient of solvents. Finally, the microtubes were kept in ultrapure water until their use. The microtube morphology resulting from these conditions was subsequently assessed using scanning electron microscopy (SEM), as depicted in Supplementary Figure S2. Notably, when the potential pulse was applied for 900 and 2000 s to deposit iron, the structures lacked a microtubular form, with many appearing broken. This suggests that an extended electrodeposition time was necessary to achieve the microtubular shape. Specifically, a sufficient charge was attained at 2500 s of iron electrodeposition, facilitating the microtube formation and subsequent closure of the openings. These findings imply a dependence of the microtubular shape on the iron electrodeposition time, with 2500 s being the optimal timeframe in this study for achieving the desired microtubular morphology.

**Figure 1:**
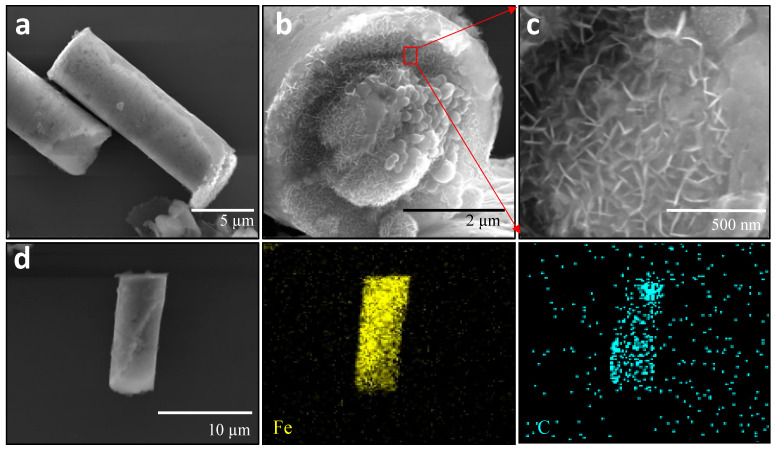
SEM images depicting the Fe-rGO micromotors from various perspectives. (**a**) The top view displays the surface morphology. (**b**) The lateral view provides a side profile (**c**) The red arrow and square denote the internal zoom view, revealing structural details. (**d**) SEM and corresponding EDX images of a Fe-rGO micromotor in mapping mode, showcasing the elemental distribution of iron (yellow) and carbon (cyan).

Figure 1a–c display representative SEM images providing different views of the Fe-rGO microtubes obtained. In Figure 1a, a distinct tubular morphology characterized by a uniform external topography can be observed. The external surface corresponds to the rGO layer, which appears thin and soft and constitutes the initial component electrodeposited in the synthesis process [32,54,58]. Figure 1b,c depict the lateral and zoomed views, revealing the internal structure of the Fe-rGO microtubes. In Figure 1c, oval-shaped nanoparticles are visible. These nanoparticles, which constitute the iron component, were consistently present in several microtubes, and their size ranged from approximately 80 to 120 nm (Supplementary Figure S3). The Fe-rGO microtubes displayed an average dimension of 14.9 ± 0.5 μm in length and 4.5 ± 0.03 μm in diameter. To further characterize the elemental composition of the Fe-rGO micromotors, SEM-EDX analysis was conducted in mapping mode. Figure 1d illustrates representative Fe-rGO SEM and EDX images, revealing a uniform distribution of iron (represented in yellow) from the core and carbon (represented in cyan) originating from the rGO throughout the microtube structure.

**Figure 2:**
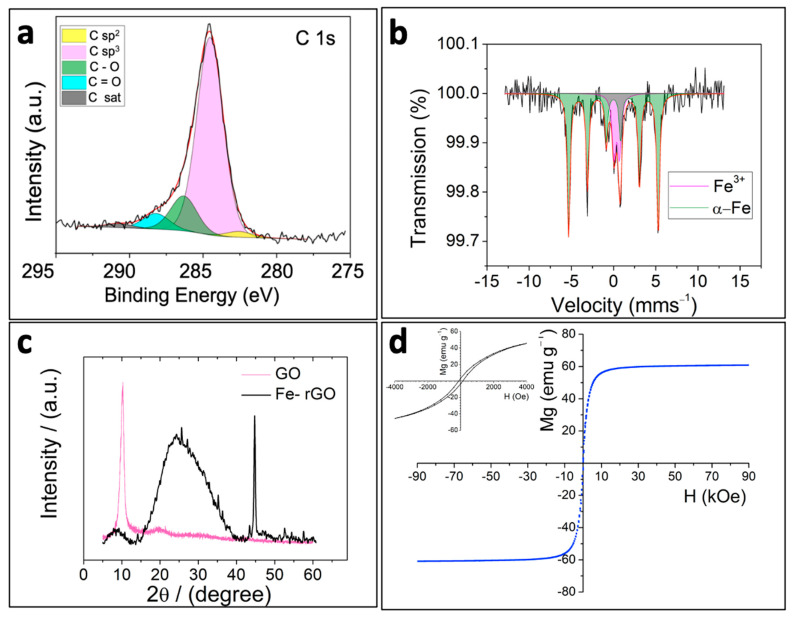
Characterization of Fe-rGO. (**a**) C1s XPS spectrum of Fe-rGO. (**b**) ^57^Fe Mössbauer spectrum of Fe-rGO. (**c**) XRD patterns comparing GO (pink) and Fe-rGO (black). (**d**) Magnetic characterization of Fe-rGO at 300 K, with inset showing a zoom of the low-field range measurements.

To determine the chemical composition of the micromotors and the iron oxidation state, the Fe-rGO micromotors were analyzed using X-ray photoelectron spectroscopy (XPS) and ^57^Fe Mössbauer, respectively. The wide-scan XPS spectrum recorded from the Fe-rGO microtubes (Supplementary Materials) was mainly dominated by the photoemission and Auger carbon peaks, while no photoemission or Auger peaks corresponding to the iron species contained in the micromotors were observed. This suggests that the iron nanoparticles were wrapped within the rGO layer like in a core–shell configuration. Figure 2a shows the XPS C 1s spectrum. It was fitted to five different components located at 282.7 eV, 284.6 eV, 286.4 eV, 288.3 eV, and 290.8 eV, which we associate with the presence of sp^2^ carbon, sp^3^ C-C, C-O, C=O, and C π-π* satellites, respectively. The spectrum very much resembles what was recorded from electrochemically reduced GO [50]. Figure 2b shows the room temperature ^57^Fe Mössbauer transmission spectra recorded from the Fe-rGO micromotors, showing two different contributions. The most intense one (green sextet), which accounts for 78% of the spectral area, has hyperfine parameters characteristic of α-Fe (isomer shift (δ) = 0.00 mms^−1^, quadrupole shift (2ε) = 0.00 mms^−1^, hyperfine magnetic field (H) = 33.0 T). The second contribution (magenta doublet), with a 22% of the spectral area, has hyperfine parameters (isomer shift (δ) = 0.38 mms^−1^, quadrupole splitting (Δ) = 0.65 mms^−1^), which are very similar to those shown by the Fe^3+^ oxyhydroxide goethite (α-FeOOH) in nanophasic form [59]. The results suggest that the micromotors are composed of an α-Fe core surrounded by a thin, nanometer-thick layer of goethite. The formation of the goethite results, most likely, from the surface oxidation of the α-Fe core, since it is well-known that nanophasic goethite is one of the corrosion products of iron under ambient conditions.

In Figure 2c, the X-ray diffraction (XRD) pattern of Fe-rGO (black pattern) is presented, and for better comparison, the XRD pattern of GO (pink pattern) has been added. The first diffraction peak observed for Fe-rGO appears at around 2θ = 8.9° with low intensity. This diffraction peak is characteristic of GO, presenting an intense signal at 2θ = 10.1° (pink pattern) [50]. However, its presence implies that the reduction of GO has been incomplete. At the same time that this GO diffraction peak decreases, another prominent and broad diffraction peak appears at 2θ = 24.6°. This prominent peak is due to the interlayer spacing decreasing from rGO layers in an amorphous state during the reduction process. On the other hand, a sharp and intensive diffraction peak is observed at 2θ = 44.8°, characteristic of zero-valent iron [60] and consistent with the ^57^Fe Mössbauer results.

Figure 2d shows the magnetic hysteresis of Fe-rGO obtained at room temperature between ±90 kOe, and the value of magnetization is given per unit of the total mass of the Fe-rGO micromotors sample. The inset is a zoom of the low-field range measurements. Fe-rGO is characterized by presenting a soft ferromagnetic behavior with H_C_ of 110 Oe (inset of Figure 2d). Remanent magnetization (M_r_) and saturation magnetization (M_s_) of 3 emu g^−1^ and 60 emu g^−1^, respectively, were obtained for the Fe-rGO. The obtained values are lower than the reported by Krajewski et al., for α-Fe nanowires (Fe-NWs) and nanoparticles (Fe-NPs) [61]. In this work, the authors reported, for Fe-NWs, Hc, Mr, and Ms values of 300 Oe, 70 emu g^−1^, and 164 emu g^−1^, respectively, while, for Fe-NPs, Hc, Mr, and Ms values of 360 Oe, 39 emu g^−1^, and 153 emu g^−1^, respectively, were obtained. Although the compared materials do not have the same composition, it is easy to note that, in any case, the as-synthesized α-Fe particles do not reach the saturation value of bulk iron (218 emu g^−1^) [40]. Clearly, in the case of the Fe-rGO, the lower value obtained for Ms is a consequence of the matrix contribution. Considering this fact, a saturation value of 218 emu g^−1^ was taken as a reference to estimate the amount of magnetic material present in the as-synthesized composite. Then, 28% of magnetic material (α-Fe and α-FeOOH) is present in the Fe-rGO.

### 3.2. In Vitro Photothermal Capacity of Fe-rGO Micromotors

**Figure 3:**
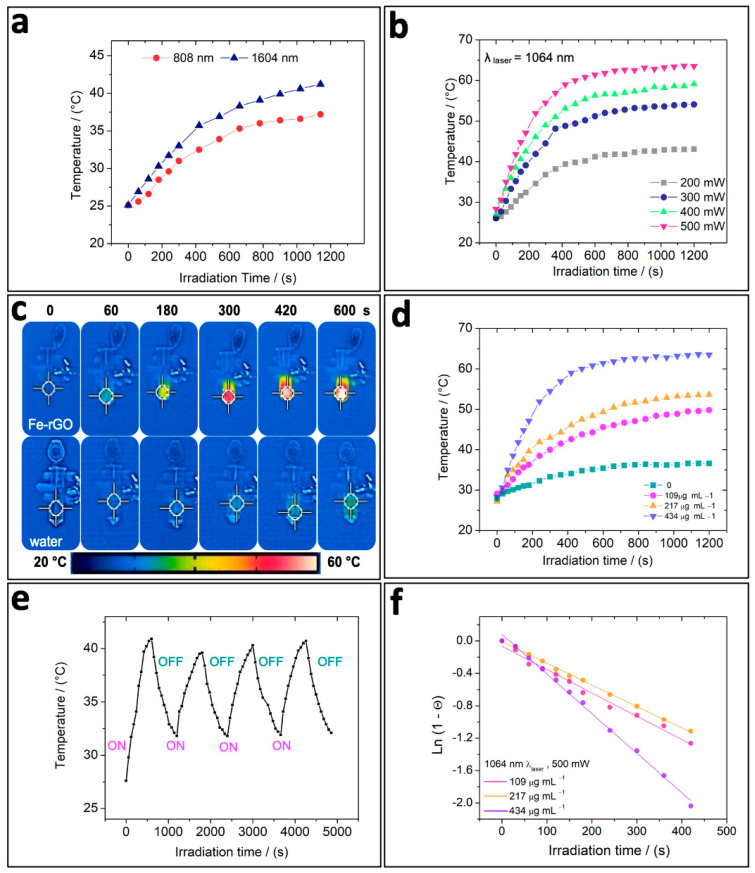
Photothermal performance of Fe-rGO micromotors dispersed in ultrapure water under laser irradiation. (**a**) Temperature change vs. irradiation time for two different laser wavelengths: 808 nm (red dotted curve) and 1064 nm (blue triangle curve). (**b**) Temperature change vs. irradiation time at various power intensities (200, 300, 400, and 500 mW). (**c**) Thermal images captured during NIR irradiation using a 1064 nm laser at 500 mW, showcasing the effect of Fe-rGO. (**d**) Photothermal heating curves at various concentrations of Fe-rGO. (**e**) Photothermal stability of Fe-rGO during 4 repeats of on/off NIR irradiation cycles. (**f**) Linear regression of Ln(1-θ) vs. time of 109 (pink), 217 (orange), and 434 µg mL^−1^; data extracted from the temperature increase in the samples using a 1064 nm laser with a power intensity of 500 mW.

To investigate the possibility of using Fe-rGO micromotors as potential photothermal agents, it was crucial to assess whether the photothermal conversion relied on the laser wavelength employed. Therefore, we examined the temperature changes during 1200 s of NIR irradiation using a standard 808 nm laser source and a 1064 nm laser source seated at a power intensity of 350 mW to irradiate Fe-rGO samples of the same concentration. It can be seen in Figure 3a that, when using a 1064 nm laser source (represented by blue triangles), higher temperatures are obtained than when using an 808 nm laser source (represented by red circles), as expected. This temperature difference is evident from the beginning of the NIR irradiation, and even when the temperature rises, it stabilizes at approximately 400 s of NIR irradiation; this is higher when using the laser wavelength from the beginning of the second window. From that point until the end of the irradiation, the ΔT was around 3.2–4.0 °C of difference. Therefore, we selected the 1064 nm wavelength as the NIR laser source to irradiate Fe-rGO micromotors from this point onwards, mostly because longer wavelengths possess less energy per photon, allowing them to penetrate deeper into tissues, due to less light scattering by bio-interfaces, causing minimal damage [52].

Then, the photothermal performance of Fe-rGO micromotors was comprehensively evaluated, using the 1064 nm laser at varying power intensities and Fe-rGO sample concentrations. Figure 3b illustrates the temperature change over 1200 s NIR irradiation at four different power intensities, ranging from 200 to 500 mW, at the same sample concentration (434 µg mL^−1^). It was evident that, as the laser power intensity increases, so does the temperature attained, suggesting a clear dependence on power intensity. Furthermore, the maximum temperature is reached between 400 and 500 s of NIR irradiation, followed by a steady state. This trend is observed regardless of the power intensity applied. Notably, when utilizing a laser power intensity of 500 mW, it is possible to obtain a ΔT of 30 °C within 7 min. Nevertheless, for the rendering of cancer cells, an equal or greater laser power intensity of 300 mW would be sufficient to cause hyperthermia after 6 min when using this system [62,63].

Figure 3c,d show the influence of the Fe-rGO micromotors on the temperature change during NIR irradiation. Figure 3c shows thermal images obtained in the absence (ultrapure water) and at a high concentration of Fe-rGO. The color of the tube containing the Fe-rGO micromotors rapidly changes when the system is exposed to NIR irradiation, due to the temperature increase. In contrast, when Fe-rGO is absent, the color scale is maintained. The temperature increase in the first case is mainly attributed to the light–heat conversion that the Fe-rGO micromotors undergo. In Figure 3d, we evaluated the Fe-rGO concentration vs. time of NIR irradiation. It can be observed that ultrapure water (green square curve) showed a non-significant increase in temperature even after 1200 s of NIR irradiation, as previously seen. In contrast, at 109, 217, and 434 µg mL^−1^ (pink, orange, and lilac curves, respectively) of Fe-rGO micromotors, there was a remarkable increase in the temperature change, demonstrating the enhanced photothermal effect of Fe-rGO. This temperature grows as the concentration grows, indicating a concentration dependence. The photothermal stability of Fe-rGO was also tested through four-cycle repeated heating (laser on) and cooling (laser off) measurements, without compromising the photothermal performance significantly, since the maximum steady-state temperature remained consistent without significant variations. This behavior indicates that Fe-rGO possesses good photothermal stability and can be reusable.

Photothermal conversion efficiency (η) is an important parameter to evaluate the photothermal capacity of different photothermal agents. It has been demonstrated that graphene-based nanomaterials exhibit excellent heat conductivity and, therefore, have been considered a great potential candidate for heat transfer and hyperthermia applications [48]. In addition, previous studies have shown that rGO presents better photothermal conversion than GO under the same experimental conditions [53,55,64,65,66,67,68,69]. To determine the photothermal conversion efficiency of Fe-rGO micromotors, we followed the method outlined by Liu and colleagues [70]. Figure 3f illustrates the linear regression of Ln (1-θ) vs. time for the three concentrations of Fe-rGO analyzed. This regression allows for the calculation of the time constant for heat transfer (τS), essential for determining the photothermal conversion efficiency (see the Supplementary Materials for the detailed calculation). Subsequently, we calculated the photothermal conversion efficiency (η) of the Fe-rGO micromotors to be 11.1%, 27%, and 78% for concentrations of 109, 217, and 434 µg mL^−1^, respectively. This result demonstrates that Fe-rGO micromotors under 1064 nm laser irradiation can generate a high photothermal conversion efficiency and can be very suitable for photothermal applications.

### 3.3. Drug Loading and Release Triggered by NIR Irradiation

**Figure 4:**
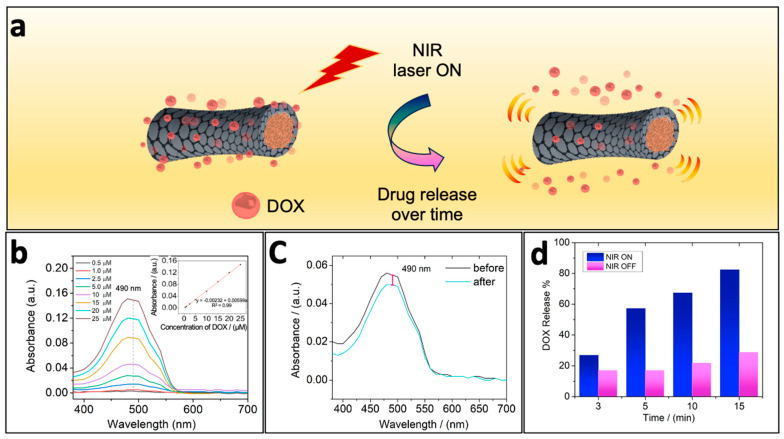
(**a**) Scheme of drug loading and release capacity from Fe-rGO micromotors under NIR irradiation. (**b**) UV–Vis absorption spectra of DOX at different concentrations and the linear relationship between the absorbance at 490 nm and the concentration of DOX (inset). (**c**) The absorbance of the DOX solution before (black curve) and after the incubation reaction (light blue curve) with Fe-rGO micromotors, pink bar shows the difference. (**d**) Effect of NIR irradiation (1064 nm) on DOX release from the Fe-rGO micromotors system.

Hyperthermia can be combined with other therapeutic methods like chemotherapy through the use of different drugs. This combined therapy can synergize the cancer treatment because some tumor cells could remain viable after PTT, especially when tumors are in very deep tissues, as they may turn into a new tumor mass in the short term [71,72]. Here, doxorubicin (DOX), a conventional chemotherapeutic drug, was used as a model drug to investigate the release capacity from Fe-rGO micromotors under NIR irradiation, as schematized in Figure 4a. The DOX drug was initially adsorbed over the rGO surface, probably through π-π stacking and electrostatic interactions. In Figure 4b, we show the UV–Vis absorption of DOX in PBS 7.4 solution at different concentrations. Following the strong band at 490 nm, we constructed the standard curve (inset of Figure 4b), and a regression line was established to determine the DOX concentration. Figure 4c shows the absorbance of the DOX solution before and after the incubation reaction with Fe-rGO micromotors. Then, the loaded DOX concentration was quantified to be 0.69 µg mL^−1^ with a loading capacity of 0.3% (*w*/*w*). A higher loading capacity is expected due to the similar chemical composition between rGO and DOX, π–π aromatic rings, and electrostatic charges that facilitate direct adsorption. However, the low loading value could be attributed to the excess DOX used, the incubation’s low stirring speed, and the reaction tube’s vertical positioning. This vertical position likely caused the micromotors to settle, sterically hindering their interaction with the drug molecules. Therefore, by addressing these factors, the loading capacity should be enhanced.

As reported in previous studies, the photothermal effect can trigger the release of cancer drugs [47,49,73]. Consequently, we measured the DOX release under NIR irradiation at pH 7.4 to ensure that the release effect is mainly due to the photothermal effect. As shown in Figure 4d, the release rate was significantly higher upon NIR irradiation than without irradiation for identical study periods. Within 15 min, almost 82.3% of DOX was released from Fe-rGO micromotors, while, at the same time point, the Fe-rGO micromotors without irradiation had released only 28.6% of the adsorbed drug. Besides, at this point, the maximum photothermal effect had already been reached. Overall, considering that some drugs are responsive to pH, a lower pH could further improve the release of these from the Fe-rGO micromotor.

### 3.4. Cytotoxicity of Fe-rGO Micromotors

**Figure 5:**
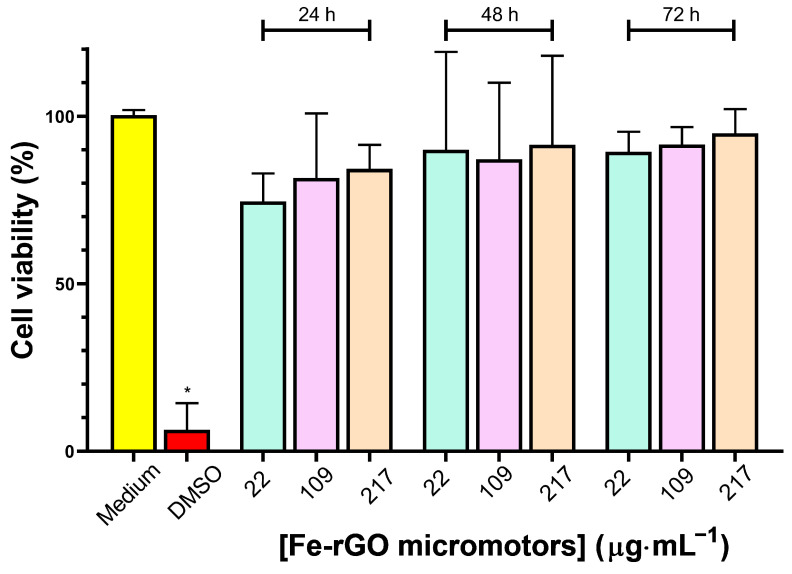
Cell viability assays based on mitochondrial metabolic activity in MDA-MB-231 breast cancer cells line at different concentrations of rGO-Fe micromotors for 24 h, 48 h, and 72 h (22 μg/mL^−1^: green; 109 μg/mL^−1^: violet; and 217 μg/mL^−1^: orange). Data are shown as means ± standard deviation from three independent experiments, with each point performed in sextuplicate. Statistical analysis was performed using a non-parametric one-way ANOVA comparing all conditions with the medium control. (*) indicate statistical significance with *p* < 0.05.

The excellent photothermal properties of Fe-rGO micromotors prompted us to perform a preliminary assay of their cytotoxicity. The concentration-dependent effect of 22 µg mL^−1^, 109 µg mL^−1^, and 217 µg mL^−1^ of Fe-rGO micromotors on the cell viability was determined via a standard MTT assay on MDA-MB-231 breast cancer cells treated for 24 h, 48 h, and 72 h (Figure 5). As shown in Figure 5, mitochondrial metabolic activity, assessed using the MTT viability assay, showed no significant difference compared to the control in the concentration range and incubation times evaluated. Overall, the effects on the viability after treatment with Fe-rGO micromotors were not significant under the conditions studied. On the other hand, we evaluated the effects on cell viability in another cell line (SH-SY5Y cells), which are cells of neuronal origin. We did not observe changes in the cell morphology by optical microscopy images carried out with Fe-rGO micromotors at 109 µg mL^−1^ for 24 h (further details in the Supplementary Materials, Figure S6). Figure S6 shows that SH-SY5Y cells, with or without the addition of Fe-rGO micromotors, maintained their morphology and normal neuritic processes (projections between neuronal cells) in culture [74,75], indicating that they remained viable. Nevertheless, to complete the cytotoxicity study, cell viability assays in healthy cell lines should be carried out in the future.

**Figure 6:**
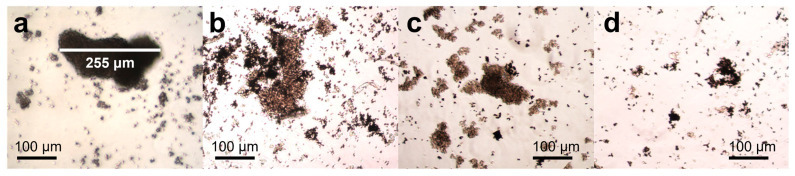
Representative and comparative images of 3D breast cancer model cells captured without micromotors (**a**) and with Fe-rGO micromotors for 96 h at different concentrations, namely (**b**) 22 µg mL^−1^, (**c**) 109 µg mL^−1^, and (**d**) 217 µg mL^−1^. Scale bar: 100 μm.

In addition, tumoroids or three-dimensional (3D) cellular models have been extensively studied nowadays, as they are more accurate response models of tumors than 2-dimensional (2D) cell cultures [76,77,78]. Accordingly, Fe-rGO micromotors’ response to the viability and morphological changes in MDA-MB-231 breast cancer cell tumoroids was studied. Figure 6 shows the optical microscopy images of one of the tests carried out after the exposition of different concentrations (22 µg mL^−1^, 109 µg mL^−1^, and 217 µg mL^−1^) of Fe-rGO micromotors with tumoroids for 96 h. Figure 6b shows a slight reduction, approximately 5%, in the effective area of the tumoroids when a concentration of 22 µg mL^−1^ was used. In contrast, Figure 6c presents a 55% decrease in the effective area when the Fe-rGO concentration increases at 109 µg mL^−1^. Moreover, when the concentration of Fe-rGO micromotors was 217 µg mL^−1^, a significant decrease of up to 91% in tumoroid size was observed, as shown in Figure 6d. Thus, high concentrations of Fe-rGO micromotors and prolonged exposure times can effectively trigger the disaggregation of tumoroids without additional stimuli.

### 3.5. Motion Analysis of Fe-rGO Micromotors

The magnetic motion of Fe-rGO micromotors was evaluated in ultrapure water (Video S1), cellular medium (Video S2), and breast cancer 3D model cells (Video S3). After dispersing the micromotors into the different kinds of media, the suspension was dripped onto a pre-cleaned glass slide, and then a magnet of 267 mT approached the glass slide. During the magnet’s approach, the micromotors started to follow a unidirectional path toward the position of the magnet. The displacement per second calculated using this magnetic force in ultrapure water was 217 ± 49 µm. In contrast, in the cellular medium, the average displacement per second was considerably lower, reaching a distance traveled of 36 ± 20 µm. Once the magnet was removed, the micromotor motion stopped. Furthermore, regardless of the medium in which they were located, the micromotors tended to form a cluster when a magnetic force was applied. This can, therefore, cause their accumulation in a specific site.

**Figure 7:**
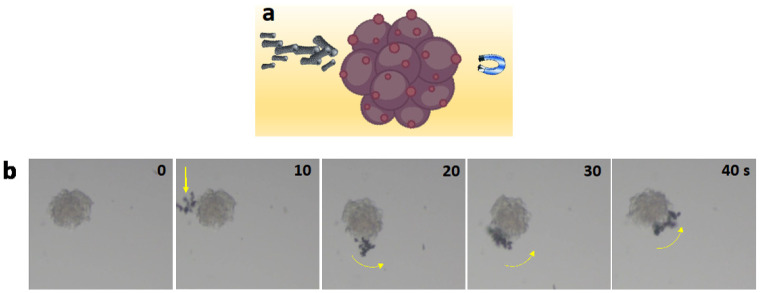
(**a**) Schematic illustration of the guided movement of Fe-rGO micromotors toward 3D cancer model cells. (**b**) Time-lapse images taken from magnetic navigation of Fe-rGO micromotors toward breast cancer 3D model cell (represented with a yellow vector) and corresponding tracking path every 10 s under the magnetic force of 267 mT (taken from Video S3, Supplementary Materials).

The guided movement of Fe-rGO micromotors toward breast cancer 3D model cells was schematically illustrated in Figure 7a. Figure 7b shows images extracted every 10 s from the time-lapse Video S3 (Supplementary Materials). It can be seen at 10 s that a cluster formed of Fe-rGO micromotors guided toward the 3D breast cancer model was formed. Then, the cluster of Fe-rGO micromotors interacted with the tumoroid, resulting in their accumulation on the tumoroid surface. Once the Fe-rGO micromotors were coupled to the 3D model cell, this interaction was maintained over time, and their direction was dependent on the direction of the magnet. The intended direction of the micromotors is shown with a yellow arrow. Additionally, the movement did not cause any breakage between the 3D model cell and the micromotors. This strong interaction is advantageous since it allows the accumulation of the Fe-rGO micromotors, specifically on cancerous sites, to subsequently generate the photothermal effect and drug release under second NIR irradiation.

## 4. Conclusions

In conclusion, we have developed a magnetic microtubular core–shell micromotor with magnetic and photothermal functionality in this study, serving as a promising secondary NIR photothermal agent. The shell, composed of rGO, exhibits strong light absorption in the second NIR region, enhancing the photothermal effect. Meanwhile, the core, consisting of zero-valent iron nanoparticles, facilitates guided movement and enables the accumulation of these micromotors on a 3D breast cancer model cell. Comparing the effects of irradiation at different wavelengths, we found superior temperature changes in the Fe-rGO dispersion when irradiated at 1064 nm compared to 808 nm, demonstrating the advantages of irradiating with laser light from the second NIR window. Additionally, we noted that the photothermal response of Fe-rGO micromotors was dependent on laser intensity and concentration. An excellent parameter for evaluating photothermal agents is the photothermal conversion efficiency. Here, we found a remarkable photothermal conversion efficiency of 78% at a Fe-rGO concentration of 434 µg mL^−1^. Furthermore, the rGO shell also allowed the loading of the chemotherapeutic drug doxorubicin, which can be rapidly released upon NIR irradiation. Finally, preliminary results from in vitro tests on a 3D breast cancer model showed low cytotoxicity and strong accumulation of these micromotors guided by magnetic motion. These promising findings suggest that Fe-rGO core–shell micromotors are viable candidates with significant potential for enhancing cancer treatment through combined photothermal therapy. The results presented have established the groundwork to continue with future work, exploring the photothermal effect of this system on 3D or in vivo models.

## Data Availability

The raw data supporting the conclusions of this article will be made available by the authors on request.

## Supplementary Materials

The following supporting information can be downloaded at: https://www.mdpi.com/article/10.3390/pharmaceutics16070856/s1. Figure S1: electrochemical synthesis, Figure S2: SEM images of Fe-rGO micromotors from different experimental conditions, Figure S3: SEM images of rGO-Fe microtubes obtained, Figure S4: UV/vis absorbance spectra of rGO-Fe, Figure S5: schematic illustration of the photothermal measurement equipment, Figure S6: analyses of Fe-rGO micromotors effect in SH-SY5Y cells, Table S1: calculated parameters: τS, hS, and photothermal transduction efficiency (η), as well as temperature increment (Tincr.), Video S1: magnetic motion of Fe-rGO micromotors in water under magnetic force, Video S2: magnetic motion of Fe-rGO microtubular motors in a cellular medium, Video S3: magnetic motion of Fe-rGO microtubular motors approaching the 3D tumor model cell.
