# Supplementary material for: Iron-Reduced Graphene Oxide Core–Shell Micromotors Designed for Magnetic Guidance and Photothermal Therapy under Second Near-Infrared Light
Source: Pharmaceutics. 2024 Jun 25;16(7):856. doi: 10.3390/pharmaceutics16070856 (PMC11279713; doi:10.3390/pharmaceutics16070856)
Supplement: Supplementary file 1 [file pharmaceutics-16-00856-s001.zip › pharmaceutics-3035009-supplementary.pdf]

pharmaceutics

MDPI

Supplementary Materials

Iron-Reduced Graphene Oxide Core–Shell Micromotors Designed for Magnetic Guidance and Photothermal Therapy under Second Near-Infrared Light

Orlando Donoso-González [1,2], Ana L. Riveros [1,2], José F. Marco [3], Diego Venegas-Yazigi [4,5], Verónica Paredes-García [5,6], Camila F. Olguín [7], Cristina Mayorga-Lobos [2,8], Lorena Lobos-González [8,9], Felipe Franco-Campos [10], Joseph Wang [11], Marcelo J. Kogan [1,2], Soledad Bollo [1,2,12], Claudia Yañez [12,13,*] and Daniela F. Báez [2,7,*]

1. Departamento Química Farmacológica y Toxicológica, Facultad de Ciencias Químicas y Farmacéuticas, Universidad de Chile, Sergio Livingstone #1007, Independencia, Santiago 8380492, Chile; orlando.donoso@ug.uchile.cl (O.D.-G.); ana.riveros@ciq.uchile.cl (A.L.R.); mkogan@ciq.uchile.cl (M.J.K.); sbollo@ciq.uchile.cl (S.B.)
2. Advanced Center for Chronic Diseases (ACCDiS), Universidad de Chile, Sergio Livingstone #1007, Independencia, Santiago 8380492, Chile; cristina.mayorga@ug.uchile.cl
3. Instituto de Química Física Blas Cabrera, Consejo Superior de Investigaciones Científicas (CSIC), Serrano 119, 28006 Madrid, Spain; jfmarco@iqfr.csic.es
4. Departamento de Química de los Materiales, Facultad de Química y Biología, Universidad de Santiago de Chile, Libertador Bernardo O'Higgins #3363, Estación Central, Santiago 9170022, Chile; diego.venegas@usach.cl
5. Centro para el Desarrollo de La Nanociencia y la Nanotecnología (CEDENNA), Universidad de Santiago de Chile, Libertador Bernardo O'Higgins #3363, Estación Central, Santiago 9170022, Chile; vparedes@unab.cl
6. Departamento de Ciencias Químicas, Facultad de Ciencias Exactas, Universidad Andrés Bello, República 275, Santiago, Santiago 8370146, Chile
7. Escuela de Medicina, Universidad de Talca, Talca 3460000, Chile; camila.olguinc@usach.cl
8. Cellular Communication Laboratory, Center for Studies on Exercise, Metabolism and Cancer (CEMC), Institute of Biomedical Sciences (ICBM), Faculty of Medicine, University of Chile, Santiago 8380492, Chile; llobos@udd.cl
9. Center for Regenerative Medicine, Institute for Sciences and Innovation in Medicine, Facultad de Medicina, Clínica Alemana Universidad del Desarrollo, Santiago 7610658, Chile
10. Research Group in Alternative Methods for Determining Toxics Effects and Risk Assessment of Contaminants and Mixtures (RiskTox), Laboratory of Food Chemistry and Toxicology, Faculty of Pharmacy, University of Valencia, 46100 Valencia, Spain; felipe.franco@uv.es
11. Department of Nanoengineering, University of California San Diego, La Jolla, CA 92093, USA; josephwang@ucsd.edu
12. Centro de Investigación de Procesos Redox, CIPRex, Facultad de Ciencias Químicas y Farmacéuticas, Universidad de Chile, Sergio Livingstone #1007, Independencia, Santiago 8380492, Chile
13. Departamento de Química Orgánica y Fisicoquímica, Facultad de Ciencias Químicas y Farmacéuticas, Universidad de Chile, Sergio Livingstone #1007, Independencia, Santiago 8380492, Chile

* Correspondence: cyanez@ciq.uchile.cl (C.Y.); daniela.baez@utalca.cl (D.F.B.)

Citation: Donoso-González, O.; Riveros, A.L.; Marco, J.F.; Venegas-Yazigi, D.; Paredes-García, V.; Olguín, C.F.; Mayorga-Lobos, C.; Lobos-González, L.; Franco-Campos, F.; Wang, J.; et al. Iron-Reduced Graphene Oxide Core–Shell Micromotors Designed for Magnetic Guidance and Photothermal Therapy under Second Near-Infrared Light. *Pharmaceutics* **2024**, *16*, 856. https://doi.org/10.3390/pharmaceutics16070856

Academic Editors: Ana Isabel Fraguas-Sánchez, Raquel Fernández García and Francisco Bolás-Fernández

Received: 14 May 2024
Revised: 17 June 2024
Accepted: 20 June 2024
Published: date

Copyright: © 2024 by the authors. Submitted for possible open access publication under the terms and conditions of the Creative Commons Attribution (CC BY) license (https://creativecommons.org/licenses/by/4.0/).

Supplementary Figure S1

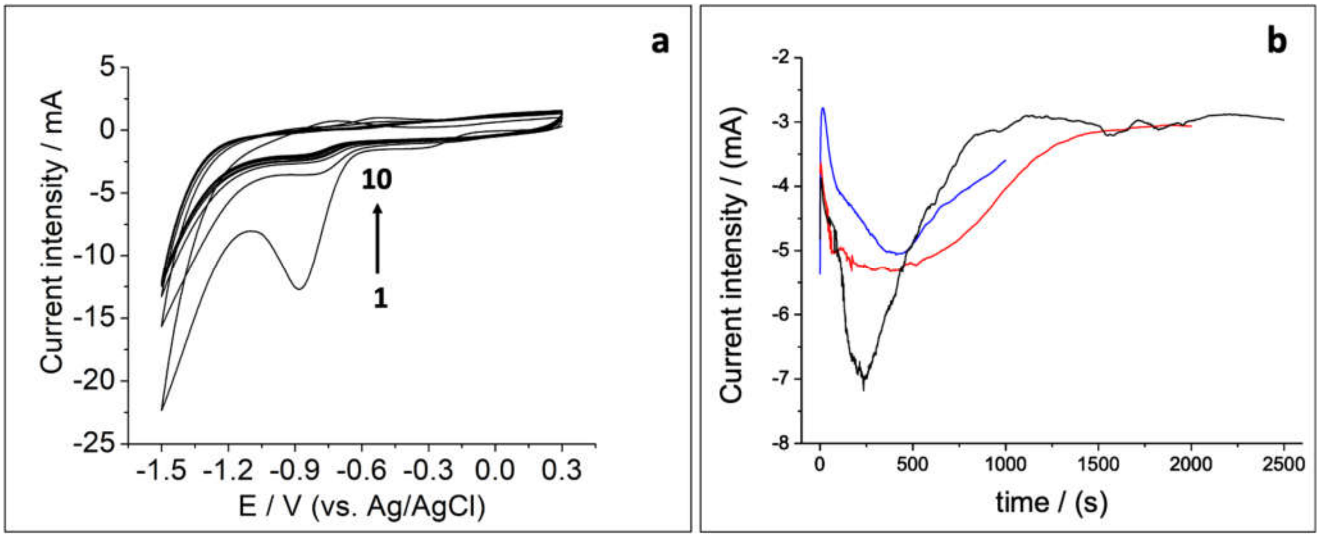

Figure S1. (a) Consecutive cyclic voltammograms at a scan rate of 50 mV s^{-1} for the reduction of graphene oxide on a 47 mm conductive polycarbonate membrane. (b) Current vs. time profile for iron deposition obtained at 900 s (blue curve), 2000 s (red curve), and 2500 s (black curve).

Supplementary Figure S2

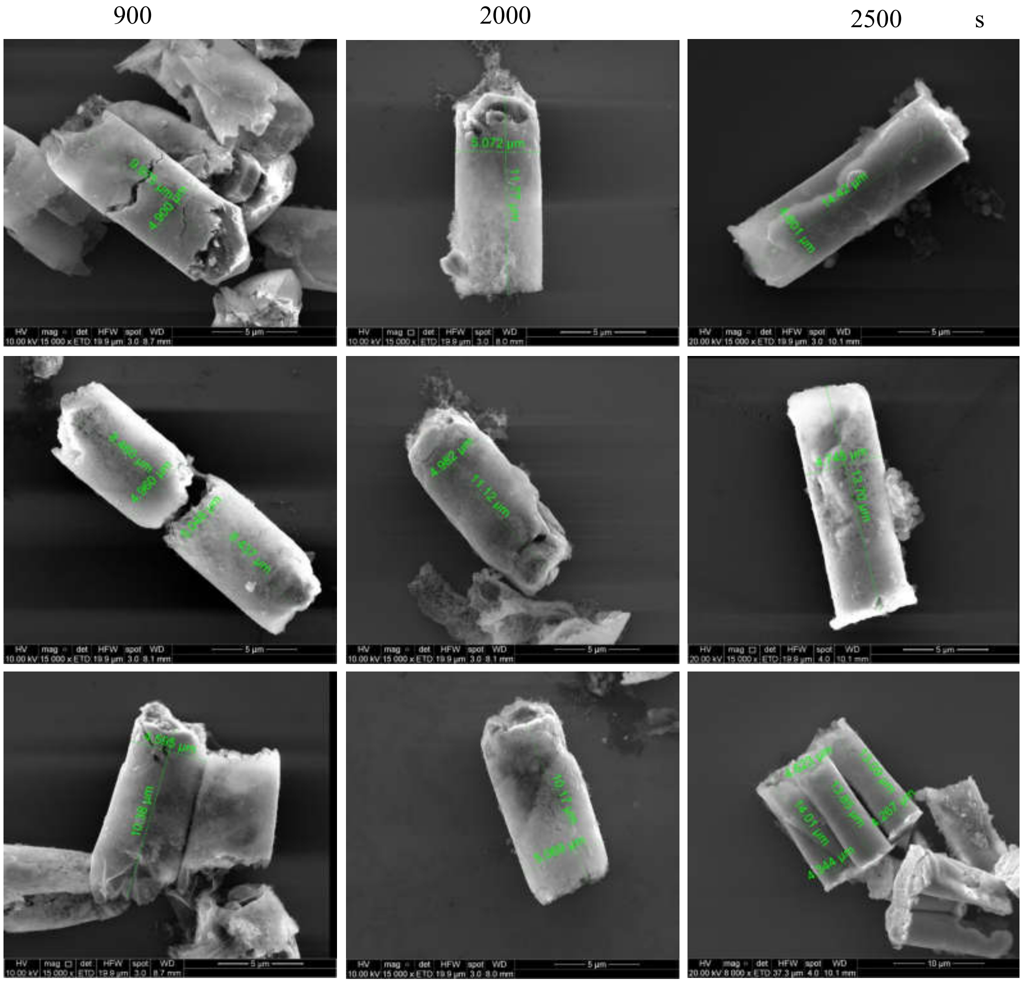

Figure S2. SEM images of rGO-Fe microtubes obtained at different times of iron electrodeposition.

Supplementary Figure S3

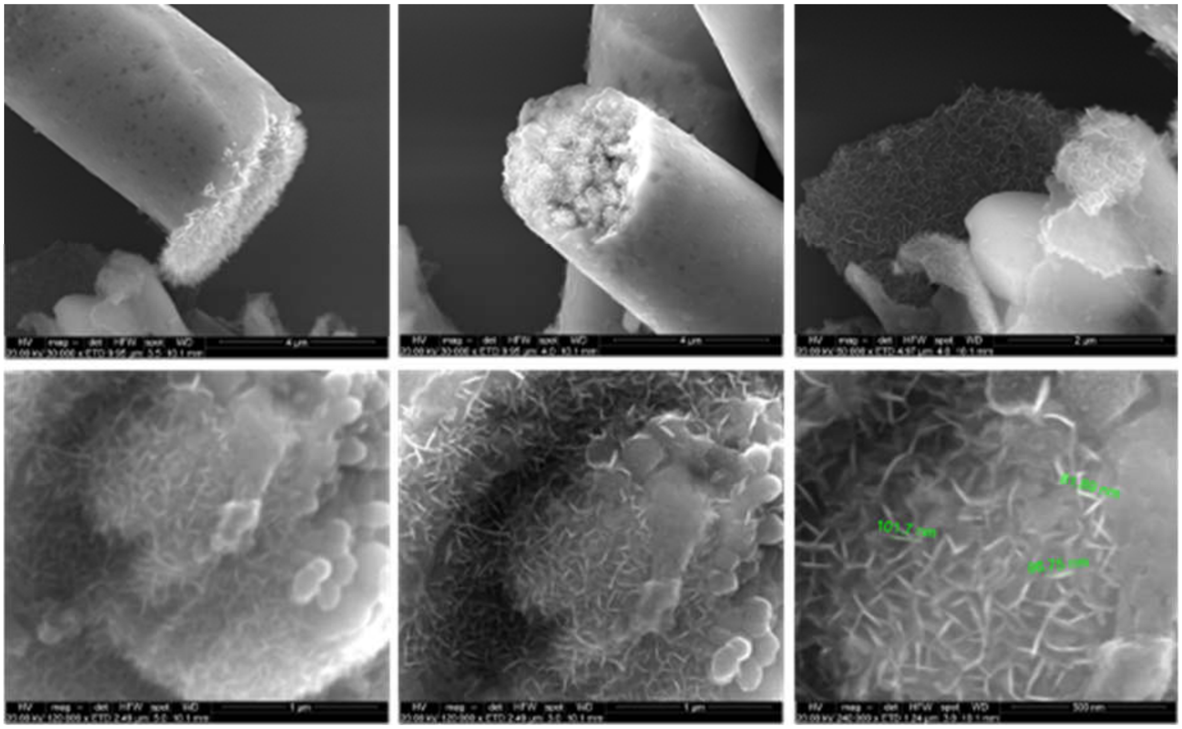

Figure S3. SEM images of rGO-Fe microtubes obtained.

Supplementary Figure S4

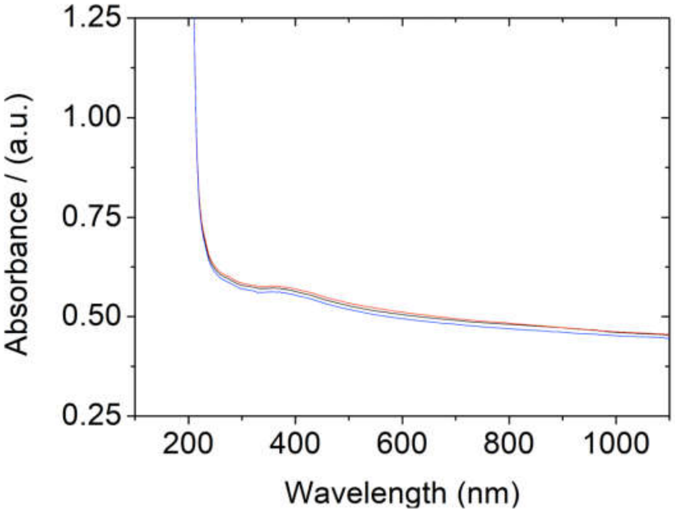

Figure S4. UV/vis absorbance spectra of rGO-Fe dispersed in ultrapure water (batch concentration).

Supplementary Figure S5

The light-to-heat energy conversion measurements were performed as illustrated in Figure S5

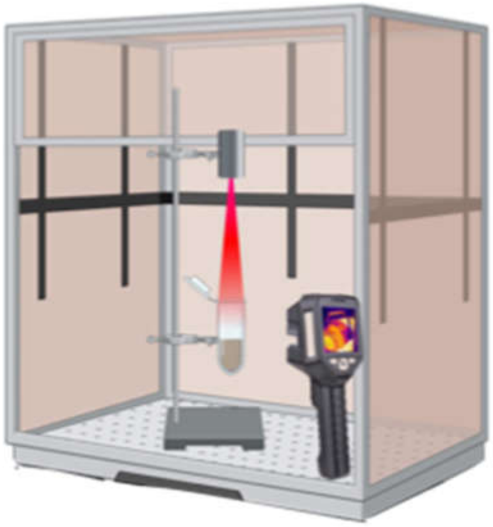

Figure S5. Schematic illustration of the equipment for the photothermal measurements of the samples. Created with biorender.

Photothermal Conversion Efficiency (η)

To calculate the photothermal conversion efficiency (η), the method described by Liu and collaborators was used[1], defining this parameter as follow:

$$\eta = \frac{hS(T_{max} - T_{room}) - Q_0}{I(1 - 10^{-A_\lambda})} \quad \text{(S1)}$$

Where h is heat transfer coefficient, S is the surface area of the container, and the value of hS is determined by working with the heating curves and introducing a dimensionless driving force theta. T_{max} and T_{room} are the equilibrium temperature of the samples and room temperature, respectively. Q_0 represents the heat dissipated from the blank, I is the laser power and A_λ is the absorbance of the rGO-Fe solution at 1064 nm.

To determinate hS, a sample system time constant τ_S was assigned in the following mass balance:

$$\tau_S = \frac{\sum_i m_i C_{p,i}}{hS} \quad \text{(S2)}$$

Then, a dimensionless driving force temperature θ was defined as follow:

$$\theta = \frac{(T_{room} - T)}{(T_{room} - T_{max})} \quad \text{(S3)}$$

Therefore, those two parameters were related by the following equation:

$$\theta = e^{(\frac{-t}{\tau_s})} \quad \text{(S4)}$$

Equation 4 can be solved by a linear regression of Ln(1-θ) vs time to obtain τ_S, as shown in Figure S6 for 109 (a), 217(b), and 434 (c) μg·mL^{-1}.

Subsequently, using the value of τ_S, and considering the mass of samples 217 μg (72% rGO, 28% Fe) and the C_P of rGO 0.710 J/g°C [1], Fe 0.450 J/g°C, and water 4.186 J/g°C, the value of hS was calculated through equation 2. Finally, using the value of hS through equation 1, the value of η was calculated. These data were summarized in Table S1.

Table S1. Calculated parameters, equation 1 and equation 2: τ_S, hS, and photothermal transduction efficiency (η); and temperature increment ($T_{incr.}$) for rGO-Fe samples at its corresponding concentration and laser power conditions.

Concentration (μg mL^{-1})	I (mW)	τ_S (s)	hS (mW/°C)	η	$T_{incr.}$ (°C)
109	500	376	11.1	11%	27.9→49.8
217	500	347	12.0	27%	27.1→53.6
434	500	245	17.0	78%	28.3→63.6

Supplementary Figure S6

Analyses of Fe-rGO micromotors effect in SH-SY5Y cells for optical microscopy images.

SH-SY5Y cells (ATCC-CRL-2266), a human neuroblastoma cell line, were cultured in DMEM/F12 medium supplemented with 10% fetal bovine serum (FBS; Biological Industries), 100 IU/mL penicillin, and 0.1 mg/mL streptomycin. The cells were incubated under pH 7.4, 5% CO2 at 37°C, and 95% air atmosphere with constant humidity. The culture medium was changed every 2–3 days. To evaluate the impact of Fe-rGO micromotors, the cells were exposed to a concentration of 109 μg/mL of these micromotors. Additionally, DMSO was utilized as a control to induce damage to the cells. Images from three independent experiments were captured using Cytation 5 (Agilent) both before and 24 hours after the addition of Fe-rGO micromotors.

The SH-SY5Y neuroblast-like cells exhibit polygonal cell bodies with short processes (Figure S6 a, b, and c) [2,3]. Figure S6 shows that both cells with and without the addition of Fe-rGO micromotors maintain their morphology and the normal processes of SH-SY5Y cells (white arrows, figure S6 d, and e), unlike cells treated with 10% DMSO for 24 hours, which exhibit harmful effects on the cells by affecting their morphology and reducing the number of projections (Figure S6 f). These results indicate that the micromotors do not have an impact on cellular viability under the studied conditions.

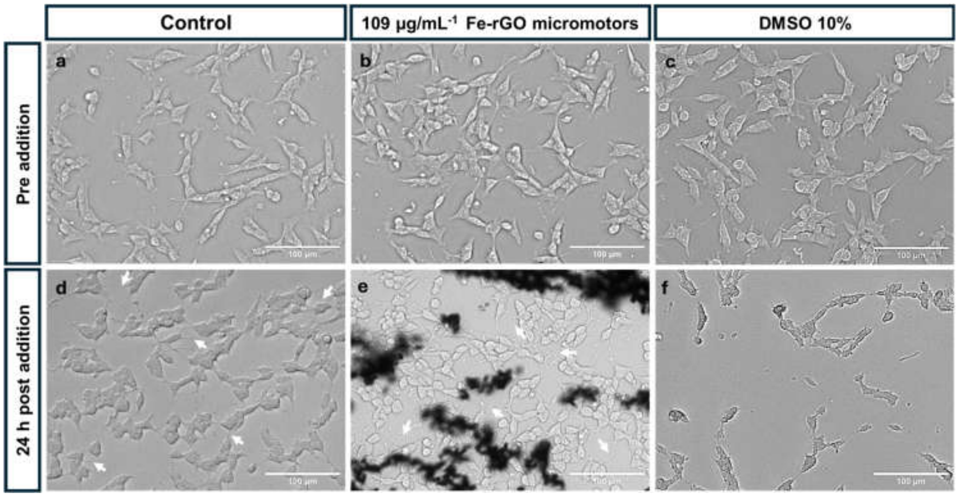

Figure S6. Representative and comparative image of SH-SY5Y cells from three independent experiment. (a and d) Control without Fe-rGO micromotors, (b and e) before and after the addition of Fe-rGO micromotors at 109 μg mL-1 for 24 h, respectively, (c and f) control before and after the addition of DMSO 10%. The white arrows indicate examples of areas with cellular processes. Scale bar: 100 μm.

References

1. Liu, W.; Zhang, X.; Zhou, L.; Shang, L.; Su, Z. Reduced Graphene Oxide (rGO) Hybridized Hydrogel as a near-Infrared (NIR)/pH Dual-Responsive Platform for Combined Chemo-Photothermal Therapy. *J. Colloid Interface Sci.* **2019**, *536*, 160–170, doi:10.1016/j.jcis.2018.10.050.
2. Lopez-Suarez, L.; Awabdh, S.A.; Coumoul, X.; Chauvet, C. The SH-SY5Y Human Neuroblastoma Cell Line, a Relevant in Vitro Cell Model for Investigating Neurotoxicology in Human: Focus on Organic Pollutants. *NeuroToxicology* **2022**, *92*, 131–155, doi:10.1016/j.neuro.2022.07.008.
3. Kovalevich, J.; Langford, D. Considerations for the Use of SH-SY5Y Neuroblastoma Cells in Neurobiology. In *Neuronal Cell Culture*; Amini, S., White, M.K., Eds.; Methods in Molecular Biology; Humana Press: Totowa, NJ, 2013; Vol. 1078, pp. 9–21 ISBN 978-1-62703-639-9.
